# The effect of floating spline parameter on the dynamic characteristic of encased differential planetary gear train

**DOI:** 10.1038/s41598-024-59094-4

**Published:** 2024-04-09

**Authors:** Xiaoyu Che, Hu Yu, Chao Zhang, Rupeng Zhu

**Affiliations:** National Key Laboratory of Science and Technology on Helicopter Transmission, Nanjing University of Aeronautics and Astronautics, Nanjing, 210016 China

**Keywords:** Encased differential planetary gear train, Load sharing characteristics, Input torque, Floating spline clearance, Spline shaft stiffness, Friction coefficient, Engineering, Physics

## Abstract

The load sharing performance of encased differential planetary system has a great impact on the operating performance and service life of the transmission system of the coaxial high speed helicopter. In order to improve the load-sharing performance of the gear pair, the influence of different floating spline structural parameters on the load sharing characteristics of the system was studied. Considering the manufacturing error, installation error, time varying meshing stiffness and other factors, the Lagrange equation is used to construct the dynamic model of encased differential planetary gear train with floating spline structure. The effects of input torque, spline clearance, spline shaft stiffness and spline friction coefficient on the load sharing performance of gear pairs were analyzed. The results show that the differential stage system has a better load sharing performance than the encased stage system. The increase of input torque helps to improve the load sharing performance of the system, and the improvement of the encased stage system is more obvious. The floating spline of sun gear of the encased stage has a greater impact on the load sharing performance of the system. Furthermore, increasing the floating spline clearance, reducing spline shaft stiffness or increasing the friction coefficient of the spline can improve the load sharing performance of the system overall.

## Introduction

Coaxial rigid rotor is an important technology for helicopters to achieve high-speed flight, but the characteristics of constant velocity reverse rotation of internal and external rotor shaft bring challenges to the structure and parameter design of the transmission system. There are currently four configurations which are cylindrical gear meshing^1–3^, bevel gear meshing^4–7^, face gear meshing^8,9^and encased differential planetary gear meshing^10^ respectively that can realize coaxial reversal output by searching for published papers and patents at home and abroad. Encased differential planetary gear train is a composite transmission configuration that couples star gear train and planetary gear train, which has the advantages of excellent bearing capacity and compact structure and has been widely used in ship engineering. However, due to the influence of the structure and the load distribution of the internal and external rotor shaft, the encased differential planetary gear train has problems such as nonuniform power distribution of the encased and differential stage system, power flow cycle and so on, resulting in its load sharing characteristics are more complex than that of star gear train or planetary gear train. In order to improve the load sharing performance of the system, it is necessary to study the load sharing structural parameters of the encased differential planetary gear train.

The transmission shaft and aeronautical gear in the helicopter main gearbox are generally connected by splines, and when the splines have radial clearance, the floating of the gear can be realized to improve the load sharing performance, which has attracted the attention of some scholars. Dong et al.^11–13^ carried out the research on the load sharing characteristics of floating spline clearance on planetary gear train, power split transmission and face gear transmission system respectively. The results show that gear floating can obviously improve the load-sharing performance. Shen et al.^14^ analyzed the influence of the internal and external spline clearance on the load sharing characteristics of the planetary gear train, but ignored the continuity of the spline support reaction force. Guo Fang et al.^15^ analyzed the effect of floating spline structure on the performance and stability of the star gear train. From the above research, it can be concluded that the floating spline structure is of great help to improve the load sharing performance of star gear train and planetary gear train, thus it can be applied to encased differential planetary gear train to enhance its load sharing performance.

The dynamic behavior of encased differential planetary gear systems have also attracted the attention of many researchers. Tan et al.^16^ conducted experimental research on encased differential planetary gearboxes with three load sharing methods: sun gear floating, flexible pin shaft and flexible inner ring gear, and explored the characteristics of encased differential planetary transmission load sharing performance with multiple load sharing measures. Hu et al.^17^ investigates the effect of random error on the dynamic load sharing characteristics of encased differential planetary gear train. Mo et al.^18^ established the nonlinear dynamic mode of encased differential planetary gear train and analyzed the effect of load torque on dynamic behavior. Zhu et al.^19^ established pure torsional freedom vibration equation of encased differential planetary train and used the multi-scale method to derive the stability conditions of summation resonance frequencies caused by the meshing stiffness fluctuations for this train. Shi et al.^20^ built a coupled dynamic model of a coaxial counter-rotating epicyclic transmission system based on the gear system dynamics and Lagrange equation, and the displacement response, speed response and the load distributions were achieved by the numerical analysis method. Kuznetsova et al.^21^ constructed the dynamic model of encased differential planetary gear train used in drilling technology and the influence of the parameters of the system on the eigenfrequencies of the oscillations is estimated. Sun et al.^22^ established a torsional-lateral dynamic model of encased differential planetary gear train and predicted the dynamic characteristics of the system.

Although scholars have carried out deep research on the dynamic behavior of encased differential planetary gear trains, there is little literature on the analysis of the load sharing behavior of encased differential planetary gear trains. Moreover, most of the research objects are single output configurations, and there is no problem of the influence of internal and external output shaft loads on the load sharing characteristics of the system, so it is necessary to conduct research on the load sharing characteristics of encased differential planetary gear trains for coaxial-counter rotating configurations. In this paper, a dynamic model with floating splines is established to analyze the effects of input torque, spline clearance, spline shaft stiffness and friction coefficient on the load sharing characteristics of the system, and provide guidance and reference for the design of floating spline structure.

## The dynamic load-sharing model of encased differential planetary gear train

### Basic structure of encased differential planetary gear train

**Figure 1:**
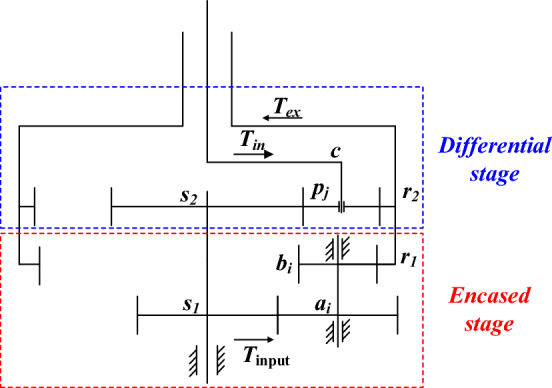
Motion diagram of encased differential planetary gear train.

The encased differential planetary gear train consists of an encased stage star gear train and a differential stage planetary gear train, and the diagram is shown in Fig. 1^23^. The encased star gear train is composed of sun gear *s*_1_, star gear *a*_i_, *b*_i_(*i* = 1, 2, …, *M*) and inner ring gear *r*_1_, and the differential stage planetary gear train is composed of sun gear *s*_2_, planetary gear *p*_j_(*j* = 1, 2,…, *N*) and inner ring gear *r*_2_. The star gear system is a fixed-axis system in which the carrier remains stationary, and the ring gear serves as the output. This configuration is utilized in the literature^24^. On the other hand, the traditional planetary gear system is a rotating system where the ring gear remains fixed, and the carrier serves as the output. This configuration is used in the literature^25^. This paper employs a differential planetary gear system, where both the ring gear *r*_2_ and the carrier *c* can rotate. However, the speeds of the sun gear *s*_2_ and the ring gear *r*_2_ can be controlled by the sun gear *s*_1_, thus achieving closed-loop motion functionality. The power transmission of the system can be divided into two paths, one is output by *r*_1_ through the encased stage system, and the other is output by *r*_2_ through the differential stage system. The constant velocity reverse output of the internal and external rotor shafts can be realized through structural parameter design.

The motion equations of the encased stage and differential stage system can be denoted by Eqs. (1), (2), respectively.

1 $$\omega_{r1} = - \frac{{z_{s1} z_{b1} }}{{z_{r1} z_{a1} }} \cdot \omega_{s1}$$

2 $$\frac{{\omega_{s2} - \omega_{c} }}{{\omega_{r2} - \omega_{c} }} = - \frac{{z_{r2} }}{{z_{s2} }}$$

where *ω*_*i*_(*i* = *s*_1_, *s*_2_, *a*_1_, *b*_1_, *r*_1_, *r*_2_) represent angular velocity corresponding to gear; *z*_*i*_(*i* = *s*_1_, *s*_2_, *a*_1_, *b*_1_, *r*_1_, *r*_2_) represent the teeth number corresponding to gear.

Furthermore, since the sun gears *s*_1_ and *s*_2_ are connected, and the ring gears *r*_1_ and *r*_2_ are connected, it follows that

3 $$\left\{ \begin{gathered} \omega_{s1} = \omega_{s2} \hfill \\ \omega_{r1} = \omega_{r2} \hfill \\ \end{gathered} \right.$$

By simultaneously solving Eqs. (1)–(3). Eq. (4) can be obtained.

4 $$\left\{ \begin{gathered} \omega_{r2} = - \frac{{z_{s1} z_{b1} }}{{z_{r1} z_{a1} }}\omega_{s1} \hfill \\ \omega_{c} = \frac{{z_{s2} z_{r1} z_{a1} - z_{r2} z_{s1} z_{b1} }}{{z_{r1} z_{a1} (z_{s2} + z_{r2} )}}\omega_{s1} \hfill \\ \end{gathered} \right.$$

To achieve the counter-rotating output of the carrier *c* and the ring gear *r*_2_, it is necessary to satisfy the condition of Eq. (5).

5 $$\omega_{c} = - \omega_{r2}$$

By simultaneously solving Eqs. (4), (5), the meshing conditions required to achieve the counter-rotating output of the encased differential planetary gear system can be obtained which can refer to Eq. (6).

6 $$\frac{{z_{s2} z_{r1} z_{a1} - z_{r2} z_{s1} z_{b1} }}{{z_{s1} z_{b1} (z_{s2} + z_{r2} )}}{ = }1$$

### Time varying meshing stiffness

Since the coincidence of gears is generally not an integer, there is a situation of alternating meshing of single and double teeth, resulting in time varying meshing stiffness, which is calculated according to the method from literature^25^, as shown in Eq. (7).

7 $$k_{m} (t) = k_{m}^{a} + 2k_{m}^{v} \sum\limits_{n = 1}^{\infty } {(a_{n} \cos n\omega t + b_{n} \sin n\omega t)}$$

where ${\text{k}}_{\text{m}}^{\text{a}}$ represents the comprehensive meshing stiffness of the gear pair *m*, $2{\text{k}}_{\text{m}}^{\text{v}}$ is the stiffness fluctuation value, *a*_n_, *b*_n_ are the Fourier transform coefficient, *ω* is the meshing period of the gear pair. The calculation of stiffness parameter values can be referred to Eqs. (8)–(11).

8 $$a_{n} = \frac{{sin(2n\pi \varepsilon_{m} )}}{n\pi }$$

9 $$b_{n} = \frac{{2[sin(n\pi \varepsilon_{m} )]^{2} }}{n\pi }$$

10 $$k_{m}^{a} = (\varepsilon_{m} - 1)k_{m}^{\max } + (2 - \varepsilon_{m} )k_{m}^{\min }$$

11 $$2k_{m}^{v} = k_{m}^{\max } - k_{m}^{\min }$$

where ${\text{k}}_{\text{m}}^{\text{min}}$ denotes the single contact stiffness, ${\text{k}}_{\text{m}}^{\text{max}}$ denotes the comprehensive meshing stiffness, *ε*_m_ denotes the contact ratio of the gear pair, which can be referred to Eq. (12)–(14).

12 $$\begin{aligned} q = & 0.04723 + \frac{0.15551}{{z_{n1} }} + \frac{0.25791}{{z_{n2} }} - 0.00635x_{1} - 0.11654\frac{{x_{1} }}{{z_{n1} }} \mp 0.00193x_{2} \\ & \quad - 0.24188\frac{{x_{2} }}{{z_{n2} }} + 0.00529x_{1}^{2} + 0.00182x_{2}^{2} \\ \end{aligned}$$

13 $$k_{m}^{min} = C_{M} C_{R} C_{B} cos\beta /q$$

14 $$k_{m}^{max} = (0.75\varepsilon + 0.25)k_{m}^{min}$$

where *z*_ni_, *x*_i_(*i* = 1, 2) represents the tooth number and modification coefficient of gear(*i* = 1 for pinion gear, *i* = 2 for bull gear), “−” and “ + ” are used for external meshing and internal meshing in the symbol “$\mp$” respectively. *C*_*M*_ denotes the theoretical correction coefficient which is generally 0.8, *C*_*R*_ is the structural coefficient of the billet which is generally 1, *C*_*B*_ is the basic tooth profile coefficient which is generally 1, *β* is the helix angle of the gear, *ε* is the contact ratio of gear pair.

The meshing damping^26^ of the gear pair can be expressed as

15 $$c_{m} = 2\xi_{m} \sqrt {\frac{{I_{m1} \cdot I_{m2} }}{{I_{m1} \cdot r_{bm2}^{2} + I_{m2} \cdot r_{bm1}^{2} }}k_{m}^{a} }$$

where *ξ*_m_ is the meshing damping ratio of the gear, the value ranges from 0.03 to 0.17, *I*_m_ is the moment of inertia (1 corresponds to the pinion gear, 2 corresponds to the bull gear), and *r*_m_ is the radius of the base circle of the gear.

### Equivalent meshing error

**Figure 2:**
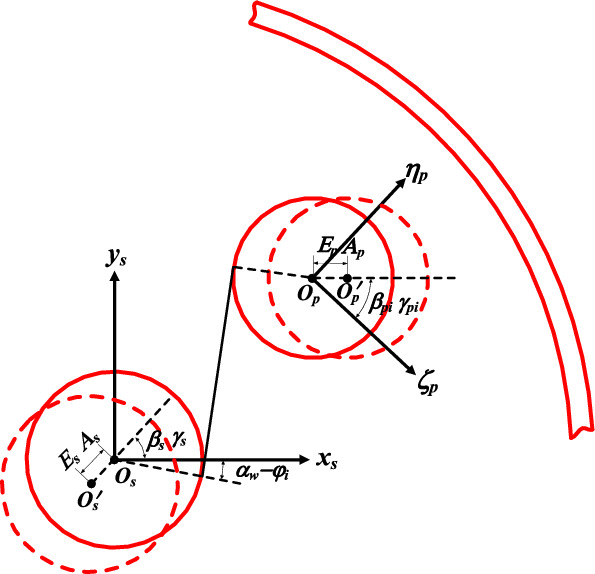
Schematic diagram of manufacturing error and installation error of the sun gear and the planetary gear.

The equivalent meshing error of the gear mainly includes manufacturing error and installation error. There are many types of manufacturing error, such as eccentricity error and tooth profile error, and this article mainly focuses on eccentricity error. Installation error refers to the error caused by the actual installation position of the gear deviating from its theoretical installation position during the installation process, and the error size is constant. Taking the planetary gear train as an example, Fig. 2 is a schematic diagram of the manufacturing error and installation error of the sun gear and the planetary gear. In the figure, the actual centroid position and the actual installation position are placed at the same point *O*_s_', *O*_p_', and the ideal centroid position and the ideal installation position are placed at the same point *O*_s_, *O*_p_.

The manufacturing and installation errors of the external meshing pair are shown as Eq. (16).

16 $$\left\{ \begin{gathered} e_{Esi} = E_{s} sin[(\omega_{s} - \omega_{c} )t + \beta_{s} + \alpha_{w} - \varphi_{i} ] \hfill \\ e_{Asi} = A_{s} sin[ - \omega_{c} t + \gamma_{s} + \alpha_{w} - \varphi_{i} ] \hfill \\ e_{Espi} = E_{p} sin[(\omega_{p} - \omega_{c} ) + \beta_{pi} + \alpha_{w} ] \hfill \\ e_{Aspi} = A_{p} sin(\gamma_{pi} + \alpha_{w} ) \hfill \\ \end{gathered} \right.$$

where *E*, *A* are the manufacturing error amplitude and the installation error amplitude respectively. *ω*_s_, *ω*_c_ are the angular velocity of the sun gear and the planetary carrier, respectively, and when the system is a fixed shaft gear train, *ω*_c_ = 0. *α*_w_ is meshing angle of the external meshing gear pair, *φ*_*i*_ is the position angle of the *i*th planetary gear relative to the initial position, which can be expressed as *φ*_i_ = 2*π*(*i* − 1)/*N*_p_ + *φ*_0_, *φ*_0_ is the initial position angle, *N*_p_ is the number of planetary gear. *β*_s_ and *γ*_s_ are the initial phase of manufacturing and installation errors of the sun gear, respectively. *β*_pi_ and *γ*_pi_ are the initial phase of manufacturing and installation errors of the planet gears.

The manufacturing and installation errors of the internal meshing pair are shown as Eq. (17).

17 $$\left\{ \begin{gathered} e_{Eri} = E_{r} sin[ - \omega_{c} t + \beta_{r} - \alpha_{n} - \varphi_{i} ] \hfill \\ e_{Ari} = A_{r} sin[ - \omega_{c} t + \gamma_{r} - \alpha_{n} - \varphi_{i} ] \hfill \\ e_{Erpi} = E_{p} sin[(\omega_{p} - \omega_{c} )t + \beta_{pi} - \alpha_{n} ] \hfill \\ e_{Arpi} = A_{p} sin(\gamma_{pi} - \alpha_{n} ) \hfill \\ \end{gathered} \right.$$

where *α*_n_ is the meshing angle of the inner meshing pair, *β*_r_, *γ*_r_ are the initial phases of manufacturing error and the installation error of the internal ring gear respectively.

The equivalent meshing error^27^ of the gear pair is

18 $$\left\{ \begin{gathered} e_{spi} = e_{Esi} + e_{Asi} + e_{Espi} + e_{Aspi} \hfill \\ e_{rpi} = e_{Eri} + e_{Ari} + e_{Erpi} + e_{Erpi} \hfill \\ \end{gathered} \right.$$

### Dynamic model of encased differential planetary gear train

The encased differential planetary gear system has (15 + 6* M* + 3*N*) degrees of freedom, and its generalized coordinates are as follow.

$$\begin{gathered} {\text{X}} = \left( {x_{{{\text{r1}}}} ,y_{{{\text{r1}}}} ,\phi_{{{\text{r1}}}} ,x_{{{\text{s1}}}} ,y_{{{\text{s1}}}} ,\phi_{{{\text{s1}}}} ,\zeta_{{{\text{ai}}}} ,\eta_{{{\text{ai}}}} ,\phi_{{{\text{ai}}}} ,\zeta_{{{\text{bi}}}} ,\eta_{{{\text{bi}}}} ,\phi_{{{\text{bi}}}} ,x_{{{\text{r2}}}} ,y_{{{\text{r2}}}} ,\phi_{{{\text{r2}}}} ,x_{{{\text{s2}}}} ,y_{{{\text{s2}}}} ,\phi_{{{\text{s2}}}} ,x_{{\text{c}}} ,y_{{\text{c}}} ,\phi_{{\text{c}}} ,\zeta_{{{\text{pj}}}} ,\eta_{{{\text{pj}}}} ,\phi_{{{\text{pj}}}} } \right)^{T} \hfill \\ \quad \quad \left( {i = {1},{2}, \ldots ,M,\;j = {1},{2}, \ldots ,N} \right) \hfill \\ \end{gathered}$$

where *r*_1_, *s*_1_, *a*_i_, *b*_i_ represent the internal gear, sun gear, first stage star gear, and second stage star gear of the encased stage system. *r*_2_, *s*_2_, *c*, *p*_j_ represent the internal gear, sun gear, carrier, and planet gear of the differential stage system. *M* and *N* represent the number of star gear and planet gear, which are both taken 3 in this paper. *x*, *y*, *φ* represent the horizontal displacement, vertical displacement, and torsional displacement of the components. *ξ*, *η* are the radial and tangential displacements of the star gear or planet gear.

The relative meshing displacement of the star gear train or planetary gear train meshing pair along the direction of the meshing line can be expressed as

19 $$\delta_{mn} = V_{mn} q_{mn} - e_{mn} (t)$$

where *V*_mn_ represents the meshing vector, with the external meshing shown in Eq. (20), and the internal meshing shown in Eq. (21). *q*_mn_ represents the degree of freedom involved in meshing, as shown in Eq. (22). *e*_mn_ represents the equivalent meshing error of the meshing pair *mn*. When the system is star gear train, *m* = *s*_1_, *r*_1_, *n* = *a*_i_、*b*_i_(*i* = 1, 2,…, *M*). When the system is planetary gear system, *m* = *s*_2_, *r*_2_, *n* = *p*_j_(*j* = 1, 2,…, *N*).

20 $$V_{mn} = \left[ {\begin{array}{*{20}l} {sin\psi_{mn} } \hfill & {\quad cos\psi_{mn} } \hfill & {\quad r_{bm} } \hfill & {\quad - sin\psi_{mn} } \hfill & {\quad - cos\psi_{mn} } \hfill & {\quad - r_{bm} } \hfill \\ \end{array} } \right]$$

21 $$V_{mn} = \left[ {\begin{array}{*{20}c} { - sin\psi_{mn} } & {\quad cos\psi_{mn} } & {\quad - r_{bm} } & {\quad sin\psi_{mn} } & {\quad - cos\psi_{mn} } & {\quad r_{bm} } \\ \end{array} } \right]$$

22 $$q_{mn} = \left[ {\begin{array}{*{20}c} {x_{m} } & {\quad y_{m} } & {\quad \phi_{m} } & {\quad \zeta_{n} } & {\quad \eta_{n} } & {\quad \phi_{n} } \\ \end{array} } \right]^{T}$$

when the meshing form is external meshing, *ψ*_mn_ = *α*_m_-*φ*_i_, the meshing form is internal meshing, *ψ*_mn_ = *α*_m_ + *φ*_i_. *α*_m_ represents the pressure angle of component *m*.

**Figure 3:**
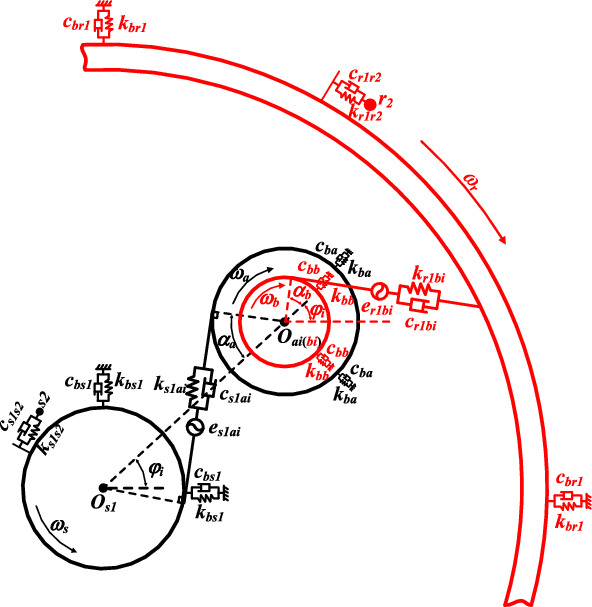
Dynamic model of encased star gear train.

**Figure 4:**
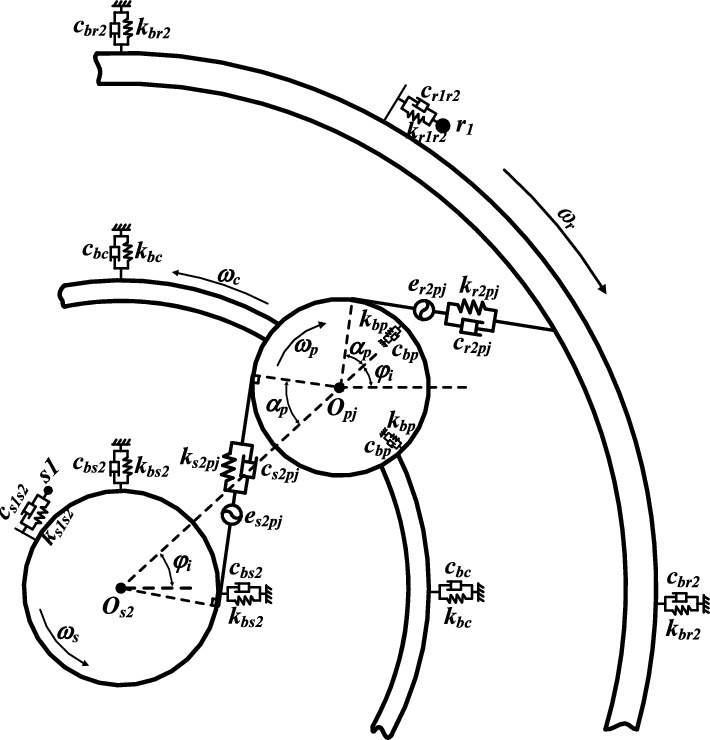
Dynamic model of differential planetary gear train.

Figures 3 and 4 show the dynamic model of the encased star gear train and the differential planetary gear train respectively. The Lagrange equation is used to derive the dynamic equation, and the derivation process can be referred to literature^28^, which is no longer mentioned in this article. The overall dynamic matrix is

$$M\left\{ {\ddot{x}} \right\} + \left( {C + \omega_{c} G} \right)\left\{ {\dot{x}} \right\} + \left( {K_{b} + K_{m} - \omega_{c}^{2} K_{\omega } } \right)\left\{ x \right\} = Q$$

where *M* is the mass matrix, *C* is the damping matrix, *G* is the gyroscopic matrix, *K*_b_ is the support stiffness matrix, *K*_m_ is the meshing stiffness matrix, *K*_ω_ is the centrifugal stiffness matrix, *Q* is the excitation vector.

### Calculation model of load sharing coefficient

Due to manufacturing error, installation errors and etc., the load distribution between the star gear or planetary gear is not equal. The *LSC*(load sharing coefficient) is usually used to describe the load distribution between the central member (sun gear, ring gear) and the planetary gear or star gear. The smaller the *LSC*, the better the load sharing performance of the system. The load sharing factor can be obtained from Eq. (23)–(24)^29^.

23 $$\left\{ \begin{gathered} b_{s1a} = {{max(M \cdot F_{s1aj} (t)} \mathord{\left/ {\vphantom {{max(M \cdot F_{s1aj} (t)} {\sum\limits_{j = 1}^{M} {F_{s1aj} (t))} }}} \right. \kern-0pt} {\sum\limits_{j = 1}^{M} {F_{s1aj} (t))} }} \, (j = 1,2,...,M{)} \hfill \\ b_{r1b} = {{max(M \cdot F_{r1bj} (t)} \mathord{\left/ {\vphantom {{max(M \cdot F_{r1bj} (t)} {\sum\limits_{j = 1}^{M} {F_{r1bj} (t)){ (}j = 1,2,...,M{)}} }}} \right. \kern-0pt} {\sum\limits_{j = 1}^{M} {F_{r1bj} (t)){ (}j = 1,2,...,M{)}} }} \hfill \\ b_{s2p} = {{max(N \cdot F_{s2pj} (t)} \mathord{\left/ {\vphantom {{max(N \cdot F_{s2pj} (t)} {\sum\limits_{j = 1}^{N} {F_{s2pj} (t))} }}} \right. \kern-0pt} {\sum\limits_{j = 1}^{N} {F_{s2pj} (t))} }} \, (j = 1,2,...,N{)} \hfill \\ b_{r2p} = {{max(N \cdot F_{r2pj} (t)} \mathord{\left/ {\vphantom {{max(N \cdot F_{r2pj} (t)} {\sum\limits_{j = 1}^{N} {F_{r2pj} (t))} }}} \right. \kern-0pt} {\sum\limits_{j = 1}^{N} {F_{r2pj} (t))} }} \, (j = 1,2,...,N{)} \hfill \\ \end{gathered} \right.$$

24 $$\left\{ \begin{gathered} LSC_{s1a} = \left| {b_{s1a} - 1} \right|_{max} + 1 \hfill \\ LSC_{r1b} = \left| {b_{r1b} - 1} \right|_{max} + 1 \hfill \\ LSC_{s2p} = \left| {b_{s2p} - 1} \right|_{max} + 1 \hfill \\ LSC_{r2p} = \left| {b_{r2p} - 1} \right|_{max} + 1 \hfill \\ \end{gathered} \right.$$

where *LSC*_s1a_, *LSC*_r1b_, *LSC*_s2p_, and *LSC*_r2p_ are the maximum *LSC*s of the sun gear-star gear pair, inner ring gear-star gear pair, sun gear-planetary gear pair and inner ring gear-planetary gear pair in a meshing cycle respectively. *F*_i_(*i* = *s*1*a*, *r*1*b*, *s*2*p*, *r*2*p*) refers to the dynamic meshing force of *i*th gear pair .

## Load sharing structure floating spline

**Figure 5:**
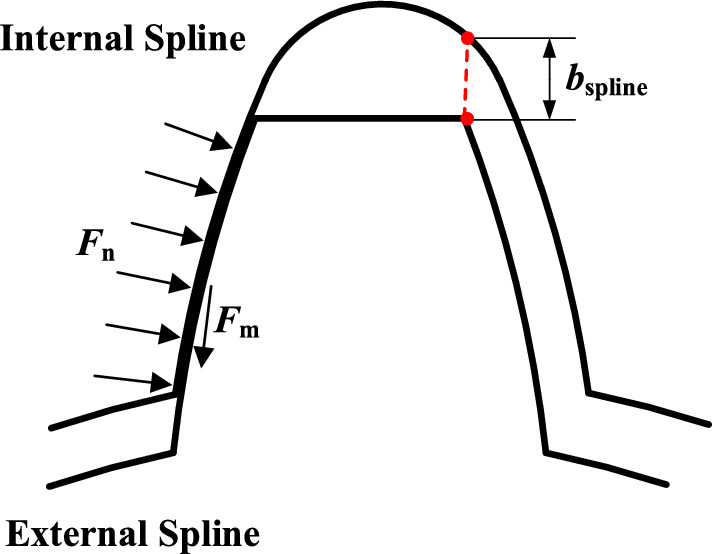
The schematic diagram of clearance between internal and external spline.

The floating spline structure is generally placed in the position of the sun gear of the encased stage system or differential stage system. When the force acting on the sun gear is unbalanced, sun gear can generate radial micro-displacements to balance the load due to the existence of radial clearance of spline connection. However, the sun gear cannot float freely resulting from the constraints of the spline shaft. The clearance between internal and external spline are shown in Fig. 5.

25 $$F_{m} = \tau \cdot F_{n}$$

where $\tau = \tau_{0} \cdot sgn(v_{s} )$, $\tau_{0}$ is the coefficient of friction, which is taken 0.1 in this paper, *v*_s_ is the relative slip velocity, *sgn*(*v*_s_ > 0) = 1, *F*_n_ is the positive pressure of the spline contact surface.

**Figure 6:**
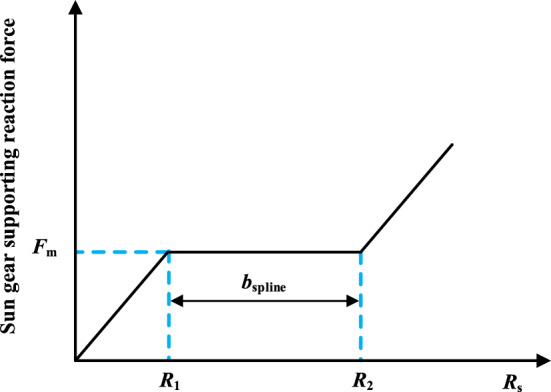
Schematic diagram of sun gear supporting reaction force with floating spline structure.

The reaction force of the spline shaft acting on the sun gear is shown in Fig. 6^30^. When 0 ≤ *R*_*s*_ < *R*_1_, the internal and external spline do not generate relative slip, and the supporting reaction force is provided by the bending deformation of the spline shaft. When *R*_1_ ≤ *R*_*s*_ < *R*_**2**_, the relative slip of the internal and external spline occurs, and the supporting reaction force is provided by spline friction force. When *R*_*s*_ ≥ *R*_**2**_, the radial clearance of the internal and external spline is eliminated, and the supporting reaction force is supported by the bending deformation of the spline shaft again. Where *R*_s_ is the floating amount of the sun gear, and *R*_1_ and *R*_2_ are the floating amount of the sun gear which undergoes the state of generating and exiting slip respectively, which can be calculated by Eq. (26)–(28).

26 $$R_{s} = \sqrt {x_{s}^{2} + y_{s}^{2} }$$

27 $$R_{1} = {{F_{m} } \mathord{\left/ {\vphantom {{F_{m} } {k_{s} }}} \right. \kern-0pt} {k_{s} }}$$

28 $$R_{2} = R_{1} + b_{spline}$$

where *k*_s_ is the bending stiffness of the splined shaft, *b*_spline_ is the radial clearance of floating spline, mm, *x*_s_ and *y*_s_ are the vibration displacements of the sun gear in the *x* and *y* directions respectively.

The function of supporting reaction force of sun gear with floating spline structure can be expressed as

29 $$F_{sx} = \left\{ \begin{gathered} k_{s} R_{s} cos\varphi { 0} \le R_{s} < R_{1} \hfill \\ F_{m} cos\varphi \, R_{1} \le R_{s} < R_{2} \hfill \\ k_{s} (R_{s} - b_{spline} )cos\varphi \, R_{s} \ge R_{2} \hfill \\ \end{gathered} \right.$$

30 $$F_{sy} = \left\{ \begin{gathered} k_{s} R_{s} sin\varphi { 0} \le R_{s} < R_{1} \hfill \\ F_{m} sin\varphi \, R_{1} \le R_{s} < R_{2} \hfill \\ k_{s} (R_{s} - b_{spline} )sin\varphi \, R_{s} \ge R_{2} \hfill \\ \end{gathered} \right.$$

where *φ* is the direction angle of the [*x*_s_, *y*_s_] vector.

## The analysis of the load-sharing characteristics about the encased differential planetary gear train

### Dynamic parameter of the encased differential planetary train

In order to conduct the dynamic analysis of encased differential planetary train, structural and dynamic parameters are needed. Table 1 shows the structural parameters of the encased differential planetary gear train, Table 2 shows the dynamic parameters of the encased differential planetary gear train^31^, Table 3 shows the error parameters of the encased differential planetary gear train, and the gear error is given according to the four-level machining accuracy^32^. This paper assumes that the output speed of the internal and external rotor shaft is 230 r/min, and the input speed of the system can be obtained according to the transmission ratio conversion and its value is 1295 r/min and the input torque is 1600 Nm.

**Table 1:** Structural parameters of encased differential planetary gear train.

Component	Tooth number	Module/mm	Pressure angle/°	Modification coefficient
*s*_1_	57	2.75	20	0.4618
*a*	54	2.75	20	0.45
*b*	18	3.5	20	0.5038
*r*_1_	107	3.5	20	0.2935
*s*_2_	38	4	20	0
*p*	25	4	20	0
*r*_2_	88	4	20	0

**Table 2:** Dynamic parameters of encased differential planetary gear train.

Dynamic parameter	Value
Support stiffness (N/m)	*k*_s_ = 3.5 × 10^8^, *k*_a_ = 2.6 × 10^8^, *k*_b_ = 3.5 × 10^8^, *k*_p_ = 5.2 × 10^8^, *k*_r_ = 6.2 × 10^8^
Torsional stiffness (Nm/rad)	*k*_ts12_ = 2.4 × 10^6^, *k*_tab_ = 8.5 × 10^5^, *k*_tr12_ = 5.7 × 10^8^
Radial coupling stiffness (N/m)	*k*_rs12_ = 2.1 × 10^8^, *k*_rab_ = 1.8 × 10^9^, *k*_rr12_ = 6.2 × 10^10^

**Table 3:** Error parameters of encased differential planetary gear trains.

Error	Sun gear	Star gear/planetary gear	Inner ring gear
*E*/mm	8	8	10
*A*/mm	± 10	± 10	± 10

### The effect of input torque on load sharing performance of encased differential planetary gear system

The input torque is a crucial parameter influencing the load-sharing characteristics of the system. However, due to the particularity of the encased differential planetary gear train structure, the torque distribution among system components differs from that of traditional planetary gear train, further impacting the load sharing behavior of the system. Therefore, it is necessary to conduct research on the torque distribution of components in the encased differential planetary gear train, followed by studying the influence of input torque on the load sharing performance of the system.

**Figure 7:**
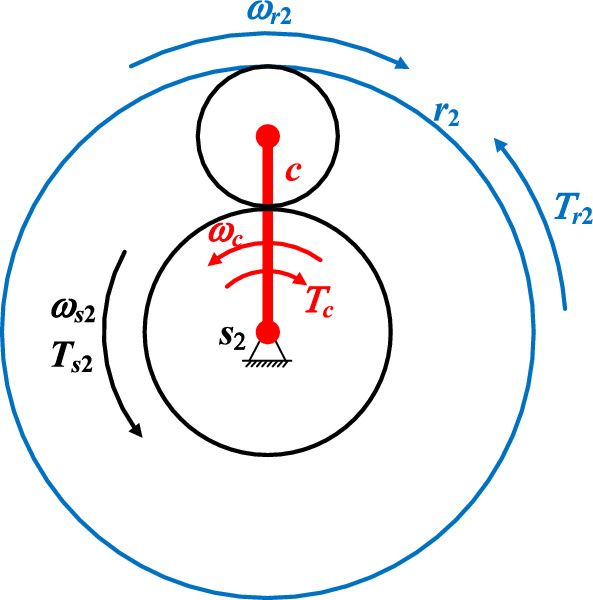
Torque diagram of differential planetary gear train.

Figure 7 illustrates the torque diagram of differential planetary gear train. Here, *ω*_*i*_ and *T*_*i*_ represent the angular velocity and torque of component *i*(*i* = *s*_2_, *c*, *r*_2_) respectively. According to literature^33^, the equilibrium relationship between torques can be obtained, as shown in Eq. (31).

31 $$\frac{{T_{s2} }}{1} = \frac{{T_{r2} }}{{ - i_{{s_{2} r_{2} }}^{c} }} = \frac{{T_{c} }}{{i_{{s_{2} r_{2} }}^{c} - 1}}$$

Assuming that the load torque ratio of the internal and external rotor shafts is *α* which can be calculated by Eq. (32).

32 $$\alpha = - \frac{{T_{in} }}{{T_{ex} }}$$

where *T*_*in*_ denotes the internal rotor shaft torque which can be expressed as *T*_*in*_ = *T*_*c*_, *T*_*ex*_ denotes the external rotor shaft torque which can be expressed as *T*_*ex*_ = *T*_*r*1_ + *T*_*r*2_, “−“ indicates that the output torque of the internal and external rotor shaft is in opposite direction. Therefore, Eq. (32) can be rewritten as

33 $$\alpha = - \frac{{T_{c} }}{{T_{r1} + T_{r2} }}$$

By simultaneously solving Eqs. (31) and (33), Eq. (34) can be obtained.

34 $$\left\{ \begin{gathered} T_{r1} = \frac{{(\alpha - 1)i_{{s_{2} r_{2} }}^{c} + 1}}{{\alpha (i_{{s_{2} r_{2} }}^{c} - 1)}}T_{c} \hfill \\ T_{r2} = \frac{{i_{{s_{2} r_{2} }}^{c} }}{{1 - i_{{s_{2} r_{2} }}^{c} }}T_{c} \hfill \\ T_{s2} = \frac{1}{{i_{{s_{2} r_{2} }}^{c} - 1}}T_{c} \hfill \\ \end{gathered} \right.$$

where $i_{s2r2}^{c}$ is the conversion transmission ratio of the differential stage system.

Since the encased stage system is a fixed-shaft gear train, the torque *T*_*s*1_ of the sun gear *s*_1_ can be expressed as

35 $$T_{s1} = T_{r1} \cdot \frac{{z_{s1} z_{b} }}{{z_{r1} z_{a} }}$$

And the torque ratio of the central components of the encased stage system to the differential stage system can be calculated by Eq. (36), (37).

36 $$\frac{{T_{s1} }}{{T_{s2} }} = \frac{{(\alpha - 1)i_{{s_{2} r_{2} }}^{c} + 1}}{\alpha } \cdot \frac{{z_{s1} z_{b} }}{{z_{r1} z_{a} }}$$

37 $$\frac{{T_{r1} }}{{T_{r2} }} = - \frac{{(\alpha - 1)i_{{s_{2} r_{2} }}^{c} + 1}}{{\alpha \cdot i_{{s_{2} r_{2} }}^{c} }}$$

**Figure 8:**
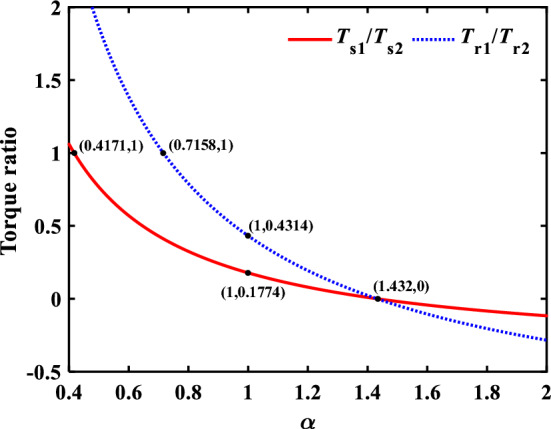
The effect of *α* on the torque ratio of the central components of the encased stage system to the differential stage system.

The effect of *α* on the torque ratio of the central components of the encased stage system to the differential stage system is shown in Fig. 8. It can be found that input torque of sun gear of encased stage and differential stage is equal when *α* = 0.4171. And the input torque of inner ring gear of encased stage and differential stage is equal when *α* = 0.7158. With the value of *α* increases to 1, *T*_s1_/*T*_s2_ = 0.1774, *T*_r1_/*T*_r2_ = 0.4314, which indicates that the input torque of central component of encased stage system is much smaller than that of differential stage system. *T*_s1_/*T*_s2_ = 0.1774, *T*_r1_/*T*_r2_ = 0.4314 when *α* = 1.432, which indicates the input power is all concentrated in the differential stage system, and the encased stage system does not bear the load. When the value of *α* is greater than 1.432, *T*_s1_/*T*_s2_ < 0 and *T*_r1_/*T*_r2_ < 0, the power flow cycle is generated inside the system, which reduces the transmission efficiency of the system in a large extent, so this situation needs to be avoided in practical applications.

**Figure 9:**
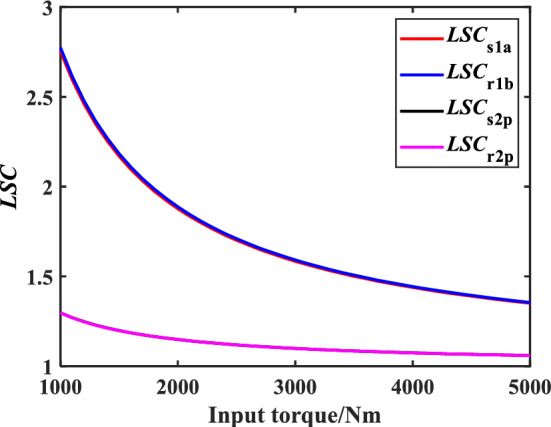
Schematic diagram of *LSC*s of gear pair of encased differential planetary gear train with the change of input torque.

Assuming that the load torques of the internal and external rotor shaft are equal, the variation law of the *LSC* of the meshing pair of the encased differential planetary gear train with the increase of input torque can be obtained, as shown in Fig. 9. It can be found that with the increase of input torque, the *LSC* of all gear pairs shows a decreasing trend, and the increase of torque inhibits the decline rate of *LSC.* Moreover, *LSC* of the encased stage gear pair is greater than that of the differential stage gear pair, and the difference of *LSC* gradually decreases with the increase of the input torque. When the input torque increases from 1000 to 5000Nm, the *LSC* of the encased stage system decreases by 51.2%, from 2.774 to 1.355, where the *LSC* of the encased stage system can be obtained by the mean value of *LSC*_s1a_ and *LSC*_r1b_, the calculation of *LSC* about the differential stage system is the same. Similarly, the *LSC* of the differential stage system decreases by 17.2%, from 1.279 to 1.059. It can be seen from Fig. 8 that torque of differential stage system is much greater than that of encased stage system when the load torque of internal and external rotor shaft is equal. This is because when the error amplitude of component is constant, the error displacement excitation projected into the direction of the meshing line is also constant, resulting in the stable error excitation load of the gear pair which can be found from Eq. (10). The meshing force of the gear pair increases as the input torque increases, which results in the influence of load distribution caused by error excitation become less, and system can achieve better load sharing performance. In summary, the load sharing performance of the differential stage system is much better than that of the closed stage system, but this gap will gradually decrease as the input torque increases. Therefore, it can be found that with the main goal of improving the load sharing performance of the encased stage system, the overall improvement of the encased differential planetary gear system is more obvious.

### The effect of floating spline clearance on load sharing performance of encased differential planetary system

**Figure 10:**
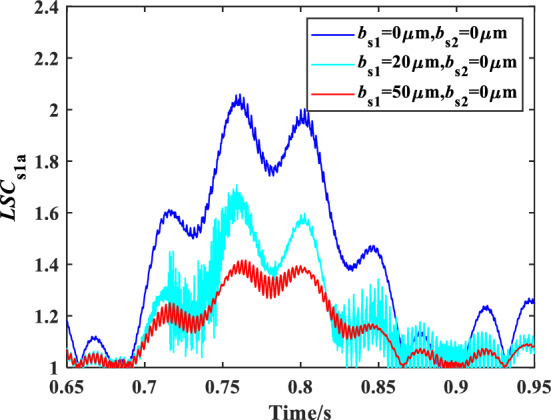
Schematic diagram of the change of *LSC* of gear pair *s*_1_*a* under different floating spline clearance.

The influence of floating spline clearance of sun gear on the load sharing characteristics of encased differential planetary gear train is studied in this section, in which the bending stiffness of the spline shaft of the encased and differential sun gear is 3.5 × 10^8^ N/m, and the friction coefficient of the spline is 0.1. Figure 10 shows the time domain variation curve of the *LSC*s of gear pair s1a under different floating spline clearances. It can be found that *LSC* exhibits time variant and reaches peak at *t* = 0.7611 s whose value is 2.06 when *b*_s1_ = 0 μm, *b*_s2_ = 0 μm, that is, there is no radial clearance between the internal and external spline of the sun gear. When *b*_s1_ = 20 μm, *b*_s2_ = 0 μm, *LSC*s are significantly reduced, and the maximum value of *LSC* is reduced to 1.709, while the vibration shows instability in partial time interval. This is because when the internal and external spline are in a relative slip state, the sun gear can adjust the radial deformation adaptively to improve the load sharing performance, but the change of friction direction and the impact caused by the small radial clearance affect the stability of the system vibration. The load sharing performance of the system is improved in a step further and the maximum value of *LSC* is also reduced to 1.416 when *b*_s1_ = 50 μm, *b*_s2_ = 0 μm. Furthermore, the system returned to a stable condition, which result from the spline clearance is large enough to avoid the shock of the internal and external spline.

**Figure 11:**
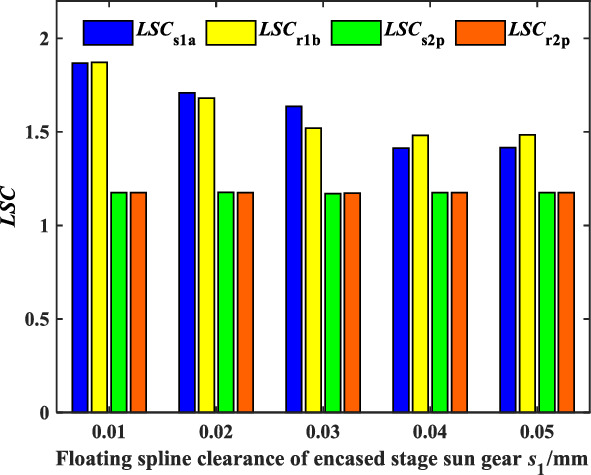
Effect of floating spline clearance of encased stage sun gear *s*_1_ on *LSC*.

**Figure 12:**
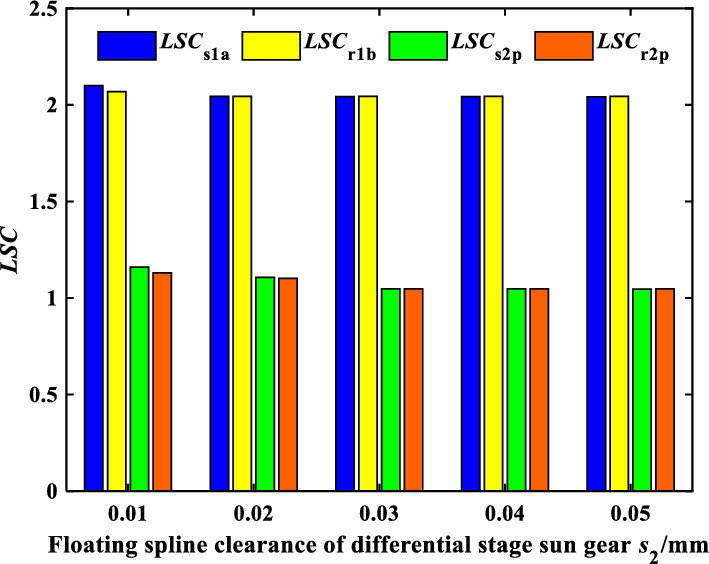
Effect of floating spline clearance of differential stage sun gear *s*_2_ on *LSC*.

Figures 11 and 12 show the effect of the spline radial clearance of the encased stage sun gear *s*_1_ and the differential stage sun gear *s*_2_ on the *LSC* of the system respectively. It can be found that the load sharing performance of the encased stage system is improved, while the load sharing performance of the differential stage system is almost unchanged when *b*_s1_ increases. When *b*_s1_ exceeds 40 μm, *b*_s1_ has no significant effect on the load sharing performance of the encased stage system. In addition, *LSC*_s1a_ > *LSC*_r1b_ when *b*_s1_ is less than 30 μm, and *LSC*_s1a_ < *LSC*_r1_ when *b*_s1_ is bigger than 30 μm conversely. It indicates that *b*_s1_ has a greater influence on the gear pair s1a than *r*_1_*b*. Moreover, the load sharing performance of both the encased stage and the differential stage system is improved a bit when the spline radial clearance of the sun gear *s*_2_ of the differential stage system increases. On the one hand, the floating spline of the sun gear of the differential stage system has little effect on the load sharing performance of the encased stage system, and on the other hand, the load sharing performance of the differential stage system is good enough. It can be concluded that adjusting the spline clearance of the encased stage sun gear *s*_1_ can improve the load sharing performance of the encased differential planetary gear train more effectively under the same condition through comparison.

### The effect of spline shaft stiffness on load sharing performance of encased differential planetary system

**Figure 13:**
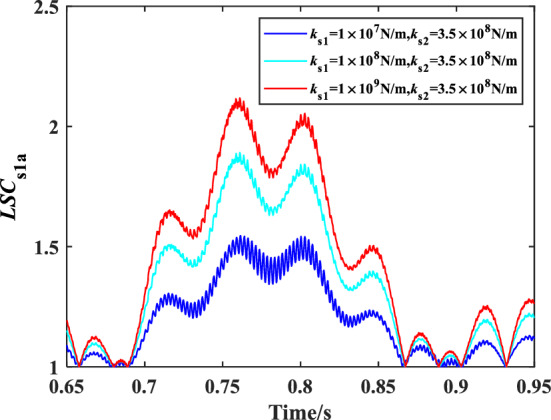
Schematic diagram of the change of *LSC*s of gear pair *s*_1_*a* under different spline shaft stiffness.

**Figure 14:**
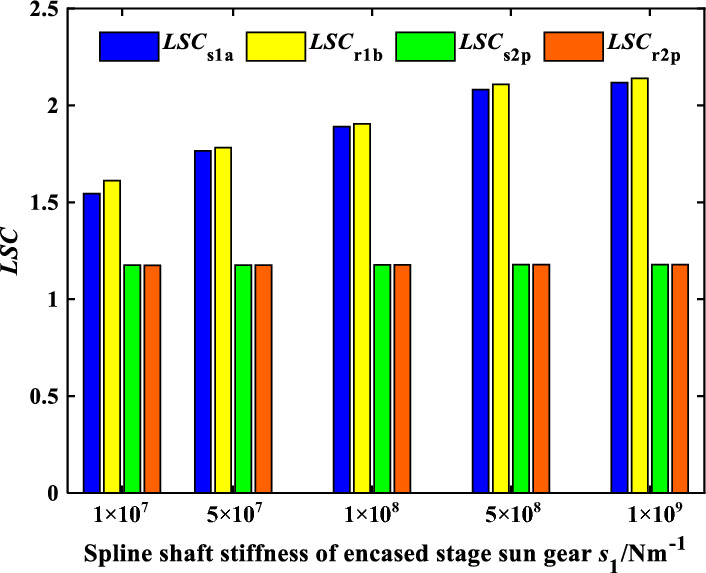
Effect of spline shaft stiffness of encased stage sun gear *s*_1_ on *LSC*.

**Figure 15:**
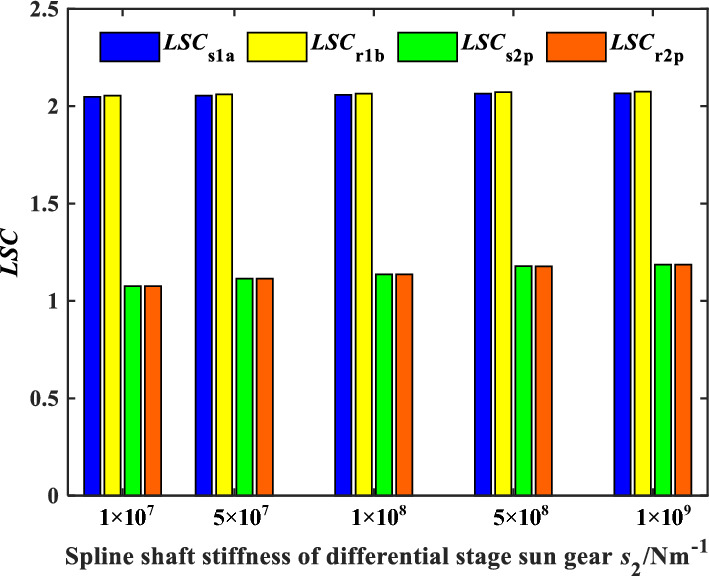
Effect of spline shaft stiffness of differential stage sun gear *s*_2_ on *LSC*.

The influence of spline shaft stiffness on the load sharing characteristics of encased differential planetary gear train is explored in this section, in which the spline clearance of the sun gear of the encased and differential stage system is 0 μm, and the friction coefficient of the spline is 0.1. Figure 13 shows the time domain variation curve of the *LSC*s of gear pair s1a under different spline shaft stiffness. It can be found that the load sharing performance of gear pair s1a is improved significantly when the spline shaft stiffness is in a condition where *k*_s2_ remains unchanged and *k*_s1_ decreases. Because the smaller the spline shaft stiffness, the larger flexibility the sun gear supporting, making it easier for the sun gear to generate floating deformation to improve the load sharing performance. Figures 14 and 15 show the effect of the spline shaft stiffness of the encased stage sun gear s1 and the differential stage sun gear s2 on the *LSC* of the system respectively. It can be found that with the increase of *k*_s1_, *LSC* of the encased stage system is increasing, while the increase rate is decreasing, and *LSC* of the differential stage system is almost unchanged. Furthermore, as the increase of *k*_s2_, *LSC*s of both the encased stage system and the differential stage system increases, while the increase amplitude is small. Therefore, the load sharing performance of the encased differential planetary gear train can be more effectively improved by reducing the spline shaft stiffness of the encased stage sun gear s1.

### The effect of friction coefficient on load sharing performance of encased differential planetary system

**Figure 16:**
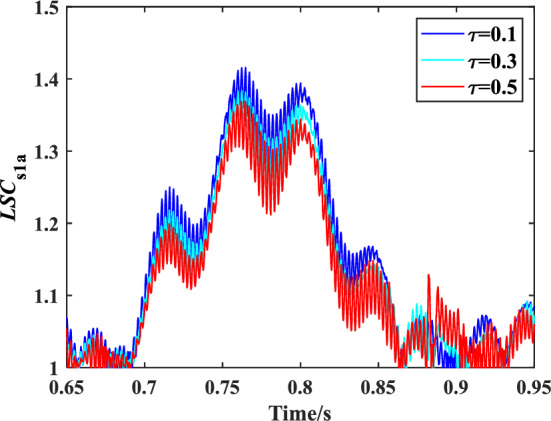
Schematic diagram of the change of *LSC* of gear pair *s*_1_*a* under different friction coefficient.

**Figure 17:**
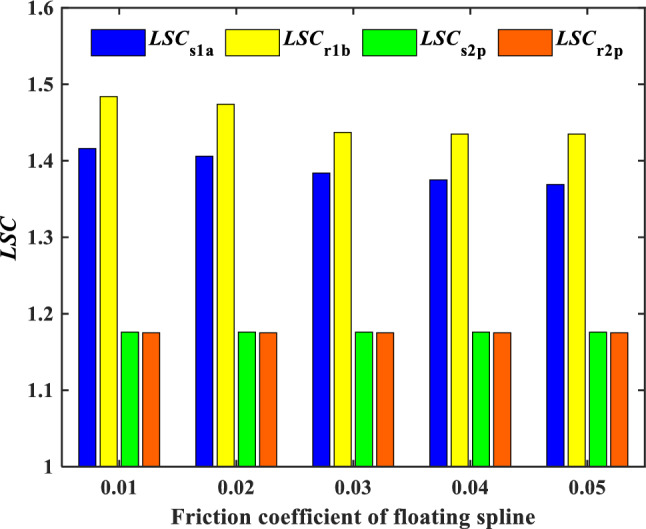
Effect of friction coefficient on *LSC*.

The influence of friction coefficient on the load sharing characteristics of encased differential planetary gear train is studied in this section, in which the spline clearance of the sun gear of the encased stage system is 50 μm, and the spline shaft stiffness is 3.5 × 10^8^N/m. Meanwhile, the spline clearance of the sun gear of the differential stage system is 0 μm, and the spline shaft stiffness is 3.5 × 10^8^ N/m. Figure 16 shows the time domain variation curve of the *LSC*s of gear pair s1a under different friction coefficient. It can be found that the load sharing performance of gear pair s1a is improved with the increase of friction coefficient. This is due to rising of friction coefficient increase *R*_1_ and *R*_2_ which can be deduced by Eqs. (21), (22). The maximum floating amount of the sun is also increased further, so the load sharing performance of the system is improved. Figure 17 shows the effect of the friction coefficient on the *LSC* of the system. It can be concluded that with the increase of friction coefficient, *LSC*s of the encased stage system is gradually decreasing, and *LSC*s of the differential stage system is almost unchanged.

### The coupling effect of sun gear spline shaft stiffness and radial clearance of the encased stage system on load sharing characteristics

**Figure 18:**
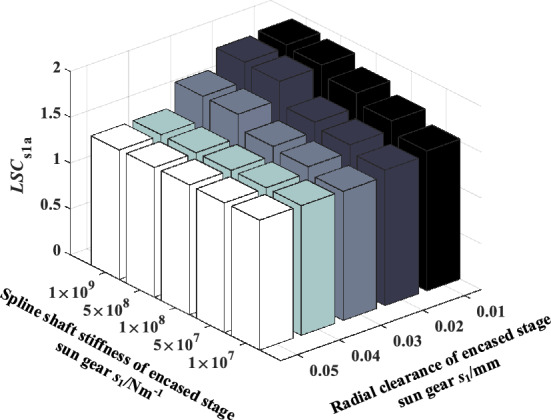
Effect of spline shaft stiffness and radial clearance of encased stage sun gear *s*_1_ on *LSC*_s1a_.

**Figure 19:**
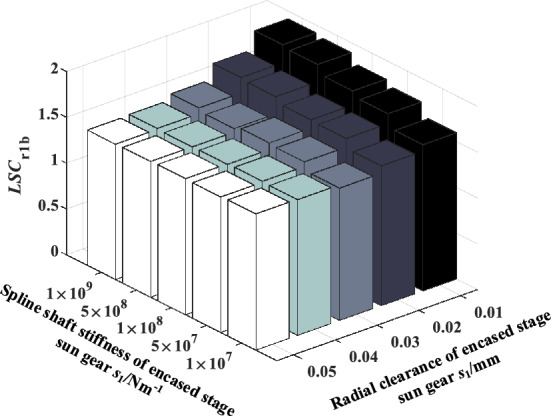
Effect of spline shaft stiffness and radial clearance of encased stage sun gear *s*_1_ on *LSC*_r1b_.

**Figure 20:**
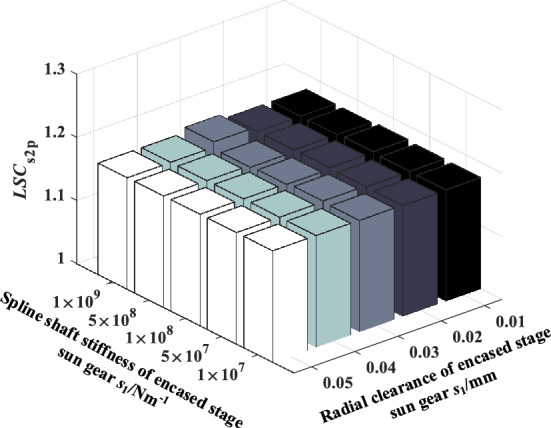
Effect of spline shaft stiffness and radial clearance of encased stage sun gear *s*_1_ on *LSC*_s2p_.

**Figure 21:**
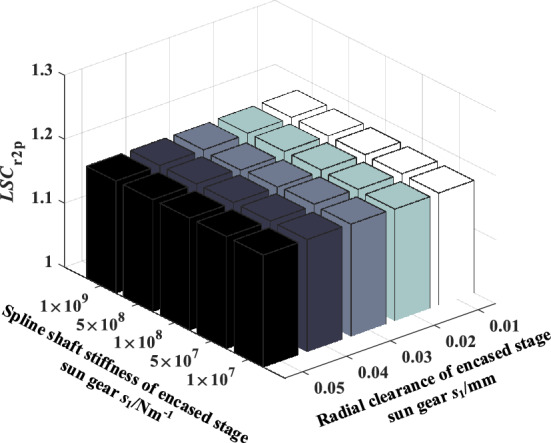
Effect of spline shaft stiffness and radial clearance of encased stage sun gear *s*_1_ on *LSC*_r2p_.

The coupling effect of spline shaft stiffness and radial clearance of the encased stage system on the load sharing performance of the system is studied in this section, in which the spline shaft stiffness of the differential stage sun gear is 3.5 × 10^8^ N/m, the spline clearance is 0 μm, and friction coefficient takes 0.1. Figures 18, 19, 20, 21 show the coupling effect of the spline shaft stiffness and radial clearance of the encased stage sun gear s1 on the *LSC*_s1a_, *LSC*_r1b_, *LSC*_s2p_ and *LSC*_r2p_ of the system respectively. It can be found that the larger spline shaft stiffness is, the more obvious the influence of radial clearance on the encased stage system. Conversely, the smaller the spline radial clearance, the more obvious the effect of spline shaft stiffness on the encased stage system. In addition, the parameters of the floating spline of the encased stage sun gear have little effect on the load sharing performance of the differential stage system.

## Conclusion

In this paper, the dynamic model of encased differential planetary gear train with floating spline is established with the consideration of the influence of time-varying mesh stiffness, manufacture error and installation error. The input torque, spline radial clearance, spline shaft stiffness and friction coefficient on the load sharing performance of the encased differential planetary gear system are studied, then the following conclusions are obtained:

1. The torque distribution of encased stage system and differential stage system are affected by the internal and external rotor shaft load torque ratio *α*. As *α* increases, both *T*_*s*1_/*T*_*s*2_ and *T*_*r*1_/*T*_*r*2_ exhibit a decreasing trend, but the rate of decrease for *T*_*r*1_/*T*_*r*2_ is faster, and the power transferred to the encased stage system decreases. When *α* exceeds 1.432, the system generates power flow cycle.
2. With the increase of input torque, the *LSC* of all gear pairs shows a decreasing trend, and the increase of torque inhibits the decline rate of *LSC.* In addition, the *LSC* of the encased stage gear pair is greater than that of the differential stage gear pair when load torque of the internal and external rotor shaft is same. While with the increase of the load torque, the difference of *LSC* is decreasing.
3. As the floating spline clearance of the encased stage sun gear *s*_1_ increases, *LSC* of the encased stage system increases, while *LSC* of the differential stage system remains almost unchanged. When the floating spline clearance of the sun gear *s*_2_ in the differential stage increases, both *LSC* of the encased stage and the differential stage systems are slightly raised. Under the same conditions, adjusting the floating spline clearance of the encased stage sun gear *s*_1_ can more effectively improve the load-sharing performance of the system.
4. With the increase of spline shaft stiffness *k*_s1_, *LSC* of the encased stage system is increasing, while the increase rate is decreasing, and *LSC* of the differential stage system is almost unchanged. In addition, as the increase of *k*_s2_, *LSC*s of both the encased stage system and the differential stage system increases, while the increase amplitude is small.
5. As the friction coefficient increases, *LSC*s of the encased stage system gradually decrease, while those of the differential stage system remain almost unchanged.

## Data Availability

The datasets generated during and/or analyzed during the current study are available from the corresponding author on reasonable request.
